# Supplementary material for: Transient rotation of photospheric vector magnetic fields associated with a solar flare
Source: Nat Commun. 2018 Jan 3;9:46. doi: 10.1038/s41467-017-02509-w (PMC5752672; doi:10.1038/s41467-017-02509-w)
Supplement: Supplementary file 2 — Description of Additional Supplementary Files [file 41467_2017_2509_MOESM2_ESM.pdf]

Description of Additional Supplementary Files

File Name: Supplementary Movie 1

Description: Time sequence of BBSO/GST azimuth maps. The image scale keeps the original resolution. In order to focus on the region of interest, a FOV of about 40'' by 40'' is selected, which is smaller than the original FOV. The cadence is about 90 s. This movie shows the changes of azimuth angle, which is defined in a ribbon-like feature, in principle moving from the right to the left. In each frame, the white contours outline the sunspot umbral areas, with magnetic flux strength greater than positive 1800 Gauss.
